# Prescribing Antidepressants and Benzodiazepines in the Netherlands: Is Chronic Physical Illness Involved?

**DOI:** 10.1155/2010/105931

**Published:** 2010-02-07

**Authors:** Jacques Th. M. van Eijk, Hans Bosma, Catharina C. M. Jonkers, Femke Lamers, Paul E. M. Muijrers

**Affiliations:** ^1^Department of Social Medicine, Faculty of Health, Medicine and Life Sciences (FHML), University Maastricht, 6200 MD Maastricht, The Netherlands; ^2^Division of Health Care, CZ Health Insurance Company, Sittard, The Netherlands

## Abstract

In this study we assessed differences in new and repeat prescriptions of psycho-tropics between patients receiving prescriptions for drugs to treat a common chronic disease and people without such prescriptions. The study used the databases of two Dutch health insurance companies (3 million people). We selected all Dutch men and women aged 45 and older who were registered for six consecutive years (1999–2004). Our analyses both found a consistent relation between psycho-tropics on the one hand and physical illness on the other. People with multi-morbidity were prescribed these drugs most often, especially men and those younger than 65. Epidemiological studies showed a prevalence of depression among people with multi-morbidity to be twice as high as among people without such conditions. According to recent guidelines non-drug treatment may be the first therapy option for patients with non severe depression. If prescribed for a long time, benzodiazepine prescriptions are especially known to be addictive. Our data raise the question to what extent patients with a chronic physical disease suffering from co-occurring mental problems are prescribed psycho-tropics in accord with the guidelines that also advise mental support in case of non severe mental problems. Further research can answer this important question.

## 1. Introduction

The expenditures on antidepressants and benzodiazepines represent a substantial burden on the Dutch health care budget [1], which also indicates that a high and growing number of patients in the Netherlands have been prescribed these drugs. It has been suggested that especially antidepressants have increasingly been prescribed for milder symptoms of depression and without receiving psychotherapy [2, 3]. Although these drugs for many patients are helpful, they also have, if prescribed inadequately, a number of adverse side effects [4]. Side effects reported for benzodiazepines include dependency, sedation, memory deficits, impaired alertness and balance, postural hypotension, risk of falling, and withdrawal reactions. A growing number of studies are reporting comparable adverse effects for antidepressants, especially among the non-severe cases [5]. Current guidelines of the Dutch and British College of General Practitioners recommend reticence in prescribing antidepressants and benzodiazepines [6–8]. The growing awareness of the adverse effects of benzodiazepines in particular has led to intervention studies being designed to help people without an indication for these drugs to end or reduce their use [9, 10]. These studies suggest that especially elderly people use these drugs against the guidelines. On the other side, it is also known that many patients, especially those with a physical condition are under diagnosed for their mental problems [11]. In an aging population, an important underlying determinant of mental health problems appears to be the presence of chronic physical illness [12].

Remarkably, existing guidelines for the diagnosis and treatment of the major chronic diseases, like arthritis, diabetes, and cardiac or pulmonary disease, appear to merely provide guidelines for the somatic disease and not for the mental problems that frequently accompany them. On the other hand, guidelines for the diagnosis and treatment of depression always mention physical disease as a possible risk factor for depressive disorder. For this reason and based on the best available evidence, the National Institute for Health and Clinical Excellence (NICE) recently published an update of the NICE recommendations regarding the treatment of depression, including a special focus on adults with a chronic physical health problem and co-occurring depression [13], emphasizing the high prevalence of mental health problems among patients with a chronic physical condition [14]. Attention to the mental health of this group of adults is becoming increasingly important, as the percentage of the population suffering from chronic degenerative diseases is expected to rise in the aging Western societies.

With regard to benzodiazepines prescriptions, guidelines recommend the use of these drugs for no longer than 6 weeks for patients without severe psychiatric problems. Antidepressants guidelines recommend prescription periods of 6–9 month or even more if recurrences show up.

Changes in the physical conditions of patients with a chronic disease constitute a long-term risk factor for mental problems in an increasing number of patients with these conditions. Instead of exclusively being prescribed pills, these patients are assumed to be much better served by strengthening their ability to cope with their burden of disease. Treatment with psychotropics conforming to the existing guidelines, is considered to be a temporal relief of the complaints [15]. Although primary care professionals are well aware of the problem, they may sometimes feel unable to do anything else than prescribe antidepressants to the category of patients with one or more chronic conditions [16]. In the Netherlands, 80% of all patients with a depressive disorder receive antidepressants as the only form of treatment, despite current recommendations for a combination of antidepressants with psychotherapy [2]. We used cross-sectional and longitudinal analysis to get more insight in the prescription of antidepressants and/or benzodiazepines in the Netherlands. Special attention will be paid to differences between patients with different chronic diseases and people without these diseases. We tried to answer the following research questions.

1. Is there an association between patient categories and the number of prescriptions for antidepressants and benzodiazepines? Are there any differences between age and sex categories?
2. What is the trend regarding new prescriptions? Are there differences between patient categories, and do these differ between age and sex categories?
3. What is the trend in repeat prescriptions of benzodiazepines? Are there any differences between patient categories?

## 2. Methods

### 2.1. Design

We carried out a *cohort study* involving 6 annual assessments for all eligible people who were registered with two insurance companies for the entire study period.

### 2.2. Study Population

The population included 734,748 Dutch men and women, aged 45 and older, who were registered with two insurance companies for the whole six-year interval between 1 January 1999 and 31 December 2004. This database only comprised people covered by compulsory health insurance under the Dutch state health insurance scheme. The database includes all prescriptions, using the Anatomical Therapeutic Chemical (ATC) Classification [17]. The numbers in the tables are different due to differences in exclusion criteria for the various analyses (see next section).

### 2.3. Assessments

**Psycho-Tropics Prescriptions**

Prescriptions of benzodiazepines and antidepressants were based on the respective ATC codes.

**Patient Classification.**

Four categories of patients were distinguished, based on prescriptions of disease-specific medication. The first category included patients continuously receiving prescriptions for diabetes, who were identified by ATC codes A10A and A10B (*n* = 11,128; 1.5% of the total population of 734.748 people). The second category received prescriptions for pulmonary disease, based on ATC codes R03A, R03B, and R03C (*n* = 20,871; 2.8%). The third category received prescriptions for cardiovascular disease, based on ATC codes C and B01 (*n* = 191,091; 26.0%). A fourth category was labelled multimorbidity, as it included patients with prescriptions for more than one of the above diseases (based on the corresponding ATC codes (*n* = 44,366; 6.0%)) To improve the validity of this patient classification, we only included only patients receiving these prescriptions in each of the six years. We assumed that patients continuously receiving these prescriptions must have the corresponding chronic condition. The remaining category was the reference category without the diseases defined above (*n* = 467,292; 63,6%  of the total population), although we cannot exclude the presence of other chronic diseases in this category.

**Demographics.**

Data about age and sex were also available in the database.

### 2.4. Analyses

Cross-sectional analyses were done to examine whether the four classes of patients based on the data of 1999 differed with regard to prescriptions for benzodiazepines and antidepressants in 1999. Logistic regression was used to compute sex- and age-adjusted odds ratios (Table 1). This was also done separately for men and women and for those younger and older than 65 (Table 2). The results section only mentions differences of more than .10 between odds ratios for the categories, although smaller differences may be also statistically significant, due to the high numbers. We then performed longitudinal analyses which excluded those who had received benzodiazepines and antidepressant prescriptions in 1999 and used the time interval until the first prescription in the 2000 to 2004 period as the outcome. These analyses were done with Cox proportional hazards analysis, which was also sex- and age-adjusted (Table 3). Analyses were again done separately for men and women and for those younger and older than 65 (Table 4). The number of years with 6–12 prescriptions for benzodiazepines was used to estimate chronic use of benzodiazepines (Table 5) [18]. People with more than 12 prescriptions in one year were also excluded from this analysis, because this might be indicative of more serious addictive disorders.

## 3. Results

### 3.1. Prescriptions for Chronic Diseases and Psychotropics

We found a clear relationship between classification and the prescriptions of benzodiazepines and antidepressants. People with medications for one of the chronic conditions were more likely to receive prescriptions for benzodiazepines and antidepressants than the reference category. The differences for benzodiazepines were somewhat clearer than those for antidepressants. We found that people with prescriptions for diabetes were largely comparable to those from the reference category, while those with medications for pulmonary and cardiovascular disease occupied an intermediate position between the reference category and those of the multimorbidity category.

Men in the multimorbidity category relatively (as compared to their reference group) had a greater chance of receiving prescriptions for benzodiazepines than their female counterparts. The same holds for prescriptions for antidepressants. People younger than 65 years with physical illness had relatively (as compared to their reference group) a greater chance of receiving benzodiazepines than older people. This was especially true for people in the multimorbidity category, and to a lesser extent for people in the pulmonary category. People in all the younger age disease categories had relatively a greater chance of receiving antidepressant medication than their older counterparts. This was especially true for people in the pulmonary category.

### 3.2. New Prescriptions for Benzodiazepines and Antidepressants

From 2000–2004, first-time prescriptions for benzodiazepines and antidepressants were provided to more than 25% and 10% of the total population, respectively. People in the pulmonary and multimorbidity categories were more likely to receive their first prescription for these medications than people in the other categories (about 8 and 4% more, resp.).

Men in the multimorbidity category had (as compared to their reference group) a greater chance of receiving benzodiazepines and antidepressants than their female counterparts. With regard to age, we found only a somewhat elevated chance for people younger than 65 years in the multimorbidity and pulmonary categories as compared to the over-65s in these categories, but only as regarding prescriptions for antidepressants.

### 3.3. Trends in Repeat Prescriptions for Benzodiazepines

People in the reference and diabetes categories generally had the lowest chance of receiving repeat benzodiazepine prescriptions, while those in the multimorbidity category were at highest risk. 

## 4. Discussion

Our cross-sectional and longitudinal analyses show a clear association between patient classification and the numbers of prescriptions for benzodiazepines and antidepressants. This was true for both new and repeat prescriptions. Men with prescriptions for physical chronic disease received more psychotropic medication than women, as were people younger than 65 compared to those older than 65, although women, in absolute terms, received more prescriptions than men. As far as new prescriptions were concerned, this was especially true for antidepressants but not for prescriptions for benzodiazepines. Chronic psychotropic prescription showed an almost linear association with classification, with persons belonging to the multimorbidity category having the lowest percentages of people without 6–12 annual prescriptions and those in the reference category having the highest. These findings are in good agreement with data recently published by LINH, a representative Dutch network of 77 general practices, showing that depressed people with comorbidity are more often prescribed benzodiazepines and antidepressants than depressed people without comorbidity [19].

Our exploratory analysis suffered from some possible pitfalls. First, we used an existing database not especially designed for this study. Therefore, we can only perform an exploration enabling the development of hypotheses for further testing. Second, we used a rather crude and indirect method to assess the presence of physical illness. We could, for example, not exclude patients with diabetic neuropathy who used antidepressants for other indications than depressive disorder. In spite of this inaccuracy, it is plausible that the trends we found are consistent and robust and thereby reassuring regarding the validity. Furthermore, as we could not select people with prescriptions for other chronic conditions, the category without prescriptions for diabetes, cardiovascular, or pulmonary disease probably comprised patients with other chronic diseases, in addition to healthy persons. Given the probable majority of people without any chronic condition in this category, however, any possible bias is assumed to be negligible and in the worst case leading to an underestimation of the associations we identified. We also tried to prevent bias by excluding sections of the total population that might contribute to invalid findings. The consistency of the findings of all our analyses regarding the difference between the categories and the comparability with the outcomes of other studies is in any case reassuring. Secondly, we used rather crude age categories. A clearer distinction between younger and older people could have made the contrast between these two categories sharper. For this reason, we controlled for age differences within the two categories. Forth, we only used the data of patients with compulsory coverage under the Dutch state health insurance scheme, who tend to belong to the lower socioeconomic groups in society. It is well-known that prescription rates for people with relatively low socioeconomic status are higher than those for people at the highest socioeconomic levels. 

Keeping these potential pitfalls in mind, our findings lead us to conclude that this analysis, with its rather robust outcome, warrants further investigation and in-depth analysis to explain the associations found and to further investigate the severity of depression and the extent to which drug treatment is combined with psychotherapy as recommended by existing guidelines in Great Britain and the Netherlands. In patients with subthreshold depression, a “wait and see” policy is preferred as well as the use of behavioural interventions as the first choice approach for patients with incident non-severe depressive and/or anxiety disorders [7, 8, 20]. The most important reason for this recommendation is the lack of effectiveness of long-term use of benzodiazepines, and to a less extent also antidepressants, in patients with mild mental problems. Although anti-depressants have proved to be as effective as psychological strategies in patients with non-severe depression, the latter seem to have better long-term effects [21]. 

Elderly people with chronic conditions seem to be a particularly neglected group, as their mental problems appear to be underdiagnosed very often in primary care [11]. Many of those, who supposedly may have been detected as having mental problems, were found to receive benzodiazepine prescriptions very frequently and exceeding a period of 6 months. Further research is needed to shed light on this probable lack of compliance with the existing guidelines. Are they too strict or might GP's feel helpless, because these patients are at permanent risk of depression or mood disturbances, or might they feel that mental problems are simply normal for the chronic condition The reason why prescribing benzodiazepines for a maximum of six weeks has become part of the guidelines is that the use of these drugs over longer periods is not helpful and even damaging, as it encourages dependency and passivity. It is worthwhile to further investigate the reason for the discrepancy between actual benzodiazepines prescription patterns and the guidelines.

In the Netherlands, there probably is an increasing tendency to treat mental problems, serious, and less serious alike, with drugs [3]. On the other hand, many patients, especially with a chronic physical disease, remain undetected with their mental problems. With the increasing prevalence of patients with a chronic physical condition, it might be worthwhile to further investigate the existing practice of the treatment of mental health problems in this group, especially in primary care. This might provide insight into possibilities to improve quality of mental health care and contribute to improvement of the guidelines for the support of patients with a chronic physical disease and mental problems.

##  Author Contributions 

Jacques Th. M. van Eijk and Paul E. M.Muijrers jointly developed the principal idea and Jacques Th. M. van Eijk drafted the paper. Jacques Th. M. van Eijk and Hans Bosma analysed the data. All authors commented on all of the previous drafts or contributed to the design of the study. All authors gave their consent to the final draft. Jacques Th. M. van Eijk is the guarantor.

## Figures and Tables

**Table 1:** Odds ratios (95% confidence interval (CI)) of receiving at least one benzodiazepines or antidepressant prescription in 1999.

	N (%)	% Men	Mean age	At least one benzodiazepines prescription (%)	Odds ratio* (95% CI) benzo-diazepines prescription	At least one anti- depressants prescription (%)	Odds ratio* (95% CI) for antidepressants prescription
Reference	467,292 (63.6)	41.8	57.5	16.5	1.00	5.9	1.00
Diabetes	11,128 (1.5)	46.0	62.2	19.2	1.15 (1.09–1.21)	6.7	1.27 (1.18–1.37)
Pulmonary	20,871 (2.8)	48.1	60.2	26.4	1.85 (1.79–1.91)	7.5	1.39 (1.32–1.47)
Cardiovascular	191,091 (26.0)	40.3	64.8	27.2	1.68 (1.66–1.71)	7.2	1.40 (1.37–1.43)
Multimorbidity	44,366 (6.0)	42.0	66.3	30.4	1.96 (1.91–2.00)	8.0	1.60 (1.54–1.66)

*Odds ratio adjusted for age and sex.

**Table 2:** Odds ratios (95% confidence interval (CI)) of receiving at least one benzodiazepines or antidepressant prescription in 1999, analysed separately for men and women and for persons younger and older than 65.

Odds ratio* (95% CI) of at least one benzodiazepines prescription			Odds ratio* (95% CI) of at least one antidepressants prescription	
	Men	Women	Men	Women
	(14.1% of 306,129	(25.0% of 428,619	(4.5% of 306,129	(7.8% of 428,619
	with prescription)	with prescription)	with prescription)	with prescription)

Reference	1.00	1.00	1.00	1.00
Diabetes	1.16 (1.07–1.26)	1.14 (1.07–1.21)	1.31 (1.15–1.49)	1.24 (1.13–1.37)
Pulmonary	1.85 (1.76–1.95)	1.91 (1.83–1.99)	1.37 (1.25–1.51)	1.43 (1.34–1.53)
Cardiovascular	1.73 (1.69–1.78)	1.66 (1.63–1.69)	1.48 (1.42–1.54)	1.36 (1.33–1.40)
Multimorbidity	2.17 (2.09–2.26)	1.87 (1.82–1.92)	1.80 (1.69–1.93)	1.53 (1.46–1.60)

	<65 years	>=65 years	<65 years	>=65 years
	(17.8% of 487,546	(25.7% of 247,202	(6.8% of 487,546	(5.8% of 247,202
	with prescription)	with prescription)	with prescription)	with prescription)

Reference	1.00	1.00	1.00	1.00
Diabetes	1.17 (1.09–1.24)	1.13 (1.05–1.21)	1.35 (1.23–1.48)	1.11 (0.98–1.26)
Pulmonary	1.97 (1.89–2.04)	1.73 (1.63–1.83)	1.52 (1.43–1.62)	1.13 (1.01–1.26)
Cardiovascular	1.72 (1.69–1.75)	1.65 (1.61–1.68)	1.50 (1.46–1.55)	1.23 (1.18–1.28)
Multimorbidity	2.15 (2.07–2.22)	1.85 (1.79–1.91)	1.83 (1.74–1.93)	1.37 (1.29–1.44)

*Odds ratio adjusted for age and sex.

**Table 3:** Hazard ratios (95% confidence interval (CI)) of benzodiazepines or antidepressant prescriptions in 2000-2004 for those without such prescriptions in 1999.

Benzodiazapines				Antidepressants		
	N	Cumulative incidence	Hazard ratio*	N	Cumulative incidence	Hazard ratio*
		(%)	(95% CI)		(%)	(95% CI)

Reference	390,050	25.1	1.00	439,658	9.3	1.00
Diabetes	8,990	26.3	1.08 (1.04–1.13)	10,384	11.1	1.25 (1.18–1.33)
Pulmonary	15,357	33.5	1.45 (1.41–1.49)	19,306	13.4	1.52 (1.46–1.58)
Cardiovascular	139,157	29.5	1.23 (1.22–1.25)	177,237	10.5	1.17 (1.14–1.19)
Multimorbidity	584,439	32.1	1.37 (1.34–1.40)	40,835	13.2	1.50 (1.46–1.55)

*Hazard ratio adjusted for age and sex.

**Table 4:** Hazard ratios (95% confidence interval (CI)) of benzodiazepines or antidepressant prescriptions in 2000–2004 for those without such prescriptions in 1999, analysed separately for men and women and for persons younger and older than 65.

	Hazard ratio* (95% CI) of incident benzodiazepines prescriptions		Hazard ratio* (95% CI) of incident antidepressants prescriptions	
	Men	Women	Men	Women
	(22.8% of 262,936 with prescription)	(30.0% of with prescription)	(8.0% of 292,267 with prescription)	(11.5% of 395,135 with prescription)
Reference	1.00	1.00	1.00	1.00
Diabetes	1.06 (1.00–1.13)	1.10 (1.04–1.16)	1.20 (1.09–1.32)	1.28 (1.19–1.38)
Pulmonary	1.44 (1.38–1.50)	1.46 (1.40–1.51)	1.48 (1.39–1.58)	1.57 (1.49–1.65)
Cardiovascul r	1.24 (1.22–1.27)	1.23 (1.21–1.25)	1.18 (1.14–1.21)	1.16 (1.13–1.18)
Multimorbidity	1.45 (1.41–1.50)	1.31 (1.27–1.35)	1.60 (1.52–1.68)	1.46 (1.41–1.51)

	< 65 years (26.4% of 400,699 with prescription)	>= 65 years (27.4% of 183,740 with prescription)	< 65 years (9.9% of 454,579 with prescription)	>= 65 years (10.3% of 232,841with prescription)

Reference	1.00	1.00	1.00	1.00
Diabetes	1.10 (1.04–1.16)	1.09 (1.02–1.16)	1.29 (1.20–1.39)	1.24 (1.13–1.36)
Pulmonary	1.44 (1.39–1.49)	1.49 (1.42–1.57)	1.57 (1.50–1.65)	1.40 (1.40–1.61)
Cardiovascular	1.22 (1.20–1.24)	1.27 (1.25–1.29)	1.20 (1.17–1.23)	1.15 (1.12–1.18)
Multimorbidity	1.36 (1.32–1.40)	1.40 (1.36–1.44)	1.60 (1.53–1.67)	1.46 (1.40–1.52)

*Hazard ratio adjusted for age and sex.

**Table 5:** Frequency distribution of people with 6–12 benzodiazepines prescriptions per year according to the number of years with these prescriptions in % (absolute numbers between brackets).

	Number of years with 6–12 prescriptions				
Grouping	0	1-2	3-4	5-6	Tot

Reference	93.8	4.0	1.2	1.0	100.0 (442.695)
Diabetes	93.4	4.2	1.3	1.2	100.0 (10.264)
Pulmonary	89.2	6.4	2.0	2.4	100.0 (18.523)
Cardiovascular	89.0	6.0	2.2	2.7	100.0 (169.764)
Multimorbidity	87.4	7.0	2.6	3.0	100.0 (37.534)

Total	92.1	4.7	1.6	1.6	100.0 (678.780)
